# In *BPS1* Downregulated Roots, the BYPASS1 Signal Disrupts the Induction of Cortical Cell Divisions in Bean-*Rhizobium* Symbiosis

**DOI:** 10.3390/genes9010011

**Published:** 2018-01-03

**Authors:** Manoj-Kumar Arthikala, Kalpana Nanjareddy, Miguel Lara

**Affiliations:** 1 Ciencias Agrogenómicas, Escuela Nacional de Estudios Superiores Unidad León—Universidad Nacional Autónoma de México (UNAM), León C.P. 37684, Mexico; manojarthik@gmail.com; 2 Instituto de Biología, Universidad Nacional Autónoma de México, Ciudad Universitaria, Coyoacan, Ciudad de México C.P. 04510, Mexico

**Keywords:** BYPASS1 gene, carotenoid biosynthesis, common bean, cortical cell divisions, infection thread, legume root nodule, *Rhizobium*

## Abstract

BYPASS1 (*BPS1*), which is a well-conserved gene in plants, is required for normal root and shoot development. In the absence of *BPS1* gene function, *Arabidopsis* overproduces a mobile signalling compound (the *BPS1* signal) in roots, and this transmissible signal arrests shoot growth and causes abnormal root development. In addition to the shoot and root meristem activities, the legumes also possess transient meristematic activity in root cortical cells during *Rhizobium* symbiosis. We explored the role of *Phaseolus vulgaris BPS1* during nodule primordium development using an RNA-interference (RNAi) silencing approach. Our results show that upon *Rhizobium* infection, the *PvBPS1-RNAi* transgenic roots failed to induce cortical cell divisions without affecting the rhizobia-induced root hair curling and infection thread formation. The transcript accumulation of early nodulin genes, cell cyclins, and cyclin-dependent kinase genes was affected in RNAi lines. Interestingly, the *PvBPS1-RNAi* root nodule phenotype was partially rescued by exogenous application of fluridone, a carotenoid biosynthesis inhibitor, which was used because the carotenoids are precursors of *BPS1* signalling molecules. Furthermore, we show that the *PvBPS1* promoter was active in the nodule primordia. Together, our data show that *PvBPS1* plays a vital role in the induction of meristematic activity in root cortical cells and in the establishment of nodule primordia during *Phaseolus-Rhizobium* symbiosis.

## 1. Introduction

The most important class of plant pigments, carotenoids, are abundant isoprenoid-derived molecules that are mainly C_40_ tetraterpenoids with a series of double bonds [1,2]. Carotenoid biosynthesis occurs in the plastids, where carotenoids are incorporated into the light-harvesting and photosynthetic reaction centre complexes. In these complexes, carotenoids serve to both absorb light energy and dissipate excess energy (photo-protection) [3,4,5]. Carotenoids can also be processed by carotenoid cleavage dioxygenases (CCDs) to form apocarotenoids, which function as hormones, flavours, and pigments, and serve as mobile signalling molecules [6,7,8,9]. 

Mobile signalling molecules are crucial to coordinate responses throughout the plant to maintain normal development. Auxins and cytokinins have been widely studied as classical long-distance signalling molecules in various aspects of plant development [10,11,12,13]. In this context, CCD7 and CCD8 are shown to be associated with the synthesis of the hormone strigolactone (SLs), a root-derived signal that inhibits shoot branching [14,15,16,17]. Strigolactones are known to be synthesized in plant roots that are colonized by arbuscular mycorrhizal (AM) fungi [7]. The roots synthesize these apocarotenoids during AM fungi symbiosis, and they are similar to those that are synthesized during carotenoid metabolism. Recent studies have shown that SLs are also found to be involved in the establishment of root nodule symbiosis in the formation of both indeterminate and determinate nodules in legumes [18,19,20,21].

Similar to SLs, BYPASS1 (*BPS1*), a putative long-distance signal, was uncovered by analysis of the *Arabidopsis BPS1* mutant. The BPS1 protein functions as a negative regulator, which is required to prevent excess production of the mobile signalling molecule. *BPS* genes are present as a family of three genes in *Arabidopsis*, all of which contribute to negative regulation of the *BPS* signal [22]. The seedlings of *BPS1* mutants constitutively produce a signal that results in failure to properly establish pro-vascular tissue, shoot and root developmental defects, and poor apical meristem activity [22]. Chimeric shoot grafting experiments in *Arabidopsis* proved that *BPS1* is a root-derived signal [23,24]. This novel signalling molecule was found to require normal carotenoid biosynthesis for its synthesis, which is conserved in plant lineages. When *BPS1* mutants are grown in media containing carotenoid biosynthesis inhibitors, such as fluridone or 2(4-chlorophenylthio)-triethylaminehydrochloride (CPTA), they become albinos due to photobleaching. However, the *BPS1* mutant phenotype of arrested root and shoot growth was partially recovered [23] when treated with inhibitors. Further analysis revealed that the *BPS* signal was not related to abscisic acid or SLs [25].

Based on a partial chemical characterization assay, Adhikari and associates [26] found that the BPS mobile molecule is a metabolite and that in *BPS1* mutants, shoot and root development is affected due to cell cycle arrest in the G_1_ phase in apical meristems. In legumes, along with the shoot and root meristem activities, transient meristematic activity occurs in root cortical cells after rhizobia infection during nodule symbiosis. Unlike other carotenoid biosynthesis pathway derived molecules abscisic acid and SLs, the role of BPS mobile signal in symbiosis is still enigmatic [27]. Root nodule symbiosis involves the development of specialized organs, called nodules, to house the symbiont, *Rhizobium*. The development of nodules requires the dedifferentiation of cortical cells at the site of infection in response to the rhizobial infection threads. Since *Arabidopsis BPS1* mutants were found to be defective in meristematic cell divisions, we hypothesized a putative role of *BPS1* in the root nodule development of legumes. To test our hypothesis, we selected *Phaseolus vulgaris BPS1* and silenced their transcripts in the hairy root system of *P. vulgaris* and analysed the nodule phenotype. We found that in *PvBPS1* downregulated roots, the BPS1 signal disrupts the induction of cortical cell divisions in bean-*Rhizobium* symbiosis.

## 2. Materials and Methods

### 2.1. Plant Growth and Rhizobium Inoculation

Seeds of the common bean (*P. vulgaris* L.) cultivar Negro Jamapa were surface sterilized and germinated in the dark for two days [28]. The two-day-old seedlings were transferred to pots with sterile vermiculite, inoculated with freshly cultured *Rhizobium tropici* (strain CIAT899) at an OD_600_ of 0.05, and irrigated with Broughton & Dilworth (B & D) [29] medium without nitrate.

### 2.2. Sequence Identification, Alignment and Phylogenetic Analysis

*Arabidopsis* BPS1 protein sequences AT1G01550.1 and AT1G01550.2 were used to retrieve the BPS1 sequences of *Lotus japonicus*, *Medicago truncatula*, *Glycine max,* and *P. vulgaris* by sequence-based homology search using BLASTP search from the genome databases *L. japonicus* gene expression atlas V2 (https://ljgea.noble.org/v2/) and Phytozome v12.1 (http://www.phytozome.net). The presence of Bypass1/DUF793 domain (PF05633) in identified sequences was verified in the *Pfam* database (http://pfam.xfam.org/) using the HMMER 3.0 program (Chevy Chase, MD, USA; http://hmmer.org/). The structures of BPS genes were analysed using the Gene Structure Display Server (http://gsds.cbi.pku.edu.cn/).

To generate the alignment of the twelve BPS1 proteins and *Arabidopsis* BPS2 and BPS3 proteins, multiple sequence alignment was performed using the T-Coffee program [30], and alignment output was generated using Boxshade version 3.21 (Köln, Germany; http://sourceforge.net/projects/boxshade/). The analyses of protein domains and conserved motifs were conducted using *Pfam* (http://www.sanger.ac.uk/software/pfam/search.html) and Multiple Em for Motif Elicitation (MEME) software [31], respectively. Phylogenetic analyses of the BPS1 proteins that are based on amino acid sequences were carried out using the Neighbour-Joining (NJ) methods in MEGA 7 [32]. NJ analyses were performed using *p*-distance methods, pairwise deletion of gaps, and the default assumptions that the substitution patterns among lineages and substitution rates among sites were homogeneous. Support for each node was tested with 1000 bootstrap replicates. Branches with bootstrap values of less than 80% were collapsed to simplify tree structures.

### 2.3. Plasmid Construction and Composite Plants

To generate the BYPASS1 promoter: β-glucuronidase (GUS) construct, a 1154-bp fragment of *PvBPS1.1* and 1124 bp of *PvBPS1.2* promoters that were upstream of the translation initiation codons were identified and isolated from *P. vulgaris* genomic DNA, according to Nanjareddy et al. [33]. The PCR fragments were cloned separately into the pENTR/D-TOPO vector (Thermo Fisher Scientific, Waltham, MA, USA) and recombined into the destination binary vector pBGWSF7.0 [34] according to the manufacturer’s instructions (Thermo Fisher Scientific, Waltham, MA, USA). To create the *PvBPS1-RNAi* construct, a fragment corresponding to the 3′-coding region of *PvBPS1* was amplified from cDNA from *P. vulgaris* root tips using specific oligonucleotides (Table S1). The PCR product was cloned into the pTdT-DC-RNAi vector [35] using the Gateway system (Thermo Fisher Scientific, Waltham, MA, USA). The resulting RNA-interference (RNAi) construct drives the transcription of a hairpin loop *PvBPS1-RNAi* under control of the 35S promoter. The pTDT-DC-RNAi vector also harbours the NOSpro:tdT cassette, which mediates the hairy root expression of the molecular fluorescent marker tdTomato [36] and allows for the identification of transformed roots. The empty pTdT-DC-RNAi vector was used as the control. The correct orientations of the above clones were confirmed by sequencing the plasmid insert. The recombinant plasmids were introduced into *Agrobacterium rhizogenes* strain K599, and then transformed into *P. vulgaris* roots using the rapid hairy root transformation method, as described recently by Nanjareddy and associates [33].

### 2.4. Physiological Analysis

Composite plants grown in pots containing sterile vermiculite were used for root growth parameters and leaf area measurement experiments. The composite plants were irrigated daily with B & D nutrient medium and maintained under growth chamber conditions with a 16-h photoperiod and 65% relative humidity at 27 ± 1 °C. Transgenic roots expressing red fluorescent protein were selected from individual plants at 10 days post-transplantation, and root growth parameters, such as root length and the lateral root density, were obtained. Lateral root density was calculated using the following formula: *D* = *LR*/*L*′, where *D* = density of lateral roots; LR = number of lateral roots; and, *L*’ = length of the main root between the first and the last lateral root [37]. Shoot parameters, such as shoot fresh weight and leaf area, were obtained from the individual *PvBPS1-RNAi* and control composite plants. The total leaf area was calculated using the Easy Leaf Area method, as described by Easlon and Bloom [38]. Composite plants grown in 15-cm glass tubes containing nutrient medium were used to observe the root hair morphology. 

### 2.5. Expression Analysis

Transcript levels were quantified using quantitative real-time PCR (RT-qPCR). The total RNA was isolated from frozen root tissues using the Plant total RNA Kit, according to the manufacturer’s recommendations (Sigma-Aldrich, St. Louis, MO, USA). To eliminate genomic DNA contamination, the RNA samples were incubated with RNase-free DNase (1 U·µL^−1^) at 37 °C for 15 min and then at 65 °C for 10 min. The RNA integrity and concentration were determined by electrophoresis and a Nanodrop ND-2000 spectrophotometer (Thermo Fisher Scientifics, Waltham, MA, USA), respectively. Quantitative real-time PCR was performed using the iScript^TM^ One-Step RT-PCR Kit with SYBR^®^ Green and an iQ5 Multicolor Real-time PCR Detection System, according to the manufacturer’s instructions (Bio-Rad, Hercules, CA, USA). Each reaction contained 40 ng of RNA as template. A control sample lacking a reverse transcriptase enzyme was included to confirm the absence of contaminant DNA. Relative expression values were calculated using the 2^−ΔCt^ method, where the quantification cycle (Cq) value equals the Cq value of the gene of interest minus the Cq value of the reference gene [39]. The *P. vulgaris* reference genes *EF1α* and *IDE* were used as internal controls [40]. The relative expression values were normalized with respect to the expression levels of these two reference genes, which were calculated according to a previously described method [41]. The reported values are the averages of three biological replicates, and each sample was assessed in triplicate. The expression levels of genes listed in Table S1 were quantified using gene-specific oligonucleotides.

### 2.6. Fluridone Treatment

The carotenoid biosynthesis inhibitor fluridone (Sigma, St. Louis, MO, USA) was applied to the roots of wild-type plants or composite plants (control and *PvBPS1-RNAi*). The wild-type plants were treated with different concentrations of fluridone (5, 10, 50, 100, and 200 µM) daily for up to ten days, and observations were taken for plant mortality. The *R. tropici*-GUS inoculated transgenic roots of composite plants were treated with 100 µM fluridone for seven days, and observations were recorded for dividing cortical cells and nodule primordia. These plants were maintained under growth chamber conditions with a 16-h photoperiod and 65% relative humidity at 27 ± 1 °C.

### 2.7. Microscopy

To analyse the rhizobial infection phenotype, control and *PvBPS1-RNAi* transgenic roots inoculated with *R. tropici*-GUS were harvested at different time points and stained for GUS activity according to Jefferson [42]. The GUS stained roots were clarified using 0.5% sodium hypochlorite for 8 h, and then examined for nodule symbiosis phenotype viz., infection threads (ITs), cortical cell divisions, nodule primordia, and nodules under a light microscope (Leica, DMLB bright-field microscope, Buffalo Grove, IL, USA). GUS-stained 18-day-old nodules were sectioned using a razor blade. The sections were mounted in 10% glycerol and observed under a light microscope. The GUS-stained transgenic roots expressing *PvBPS1.1* or *PvBPS1.2* promoters were observed under a stereo microscope (Leica), and images of nodule primordia and mature nodules were obtained. To observe the root hair morphology, uninoculated or *R. tropici*-inoculated roots were removed from the glass tubes, and the root segments were quickly mounted on microscopic slides with mounting buffer (50 mM sodium phosphate buffer pH 7.0 in 40% glycerol). The images were obtained uniformly from the root elongation zones under a light microscope.

## 3. Results

### 3.1. The BPS1 Gene Has Multiple Members

Based on *Arabidopsis BPS1* sequences (AT1G01550.1 and AT1G01550.2), the legume protein sequences were obtained from Phytozome v12.1 (https://phytozome.jgi.doe.gov/pz/portal.html) and *L. japonicus* gene expression atlas V2 (https://ljgea.noble.org/v2/). A total of 12 gene sequences were obtained from *Arabidopsis* and legumes: two genes in *Arabidopsis*, five genes in *G. max* (Glyma.10G104200.1, Glyma.09G255800.1, Glyma.07G066500.1, Glyma.18G129300.1, and Glyma.18G237000.1), two genes each in *M. truncatula* (Medtr8g028710.3 and Medtr7g078700.5) and *P. vulgaris* (Phvul.008G059500.1 and Phvul.008G059600.1), and one in *L. japonicus* (Lj1g3v2752330.1). The homologues of BPS1 were named *PvBPS1.1*, *PvBPS1.2*, etc. The predicted open reading frame coding sequences encode proteins with 184, 353, 355, 305, 351, 353, 353, 358, 353, and 347 amino acids, respectively. In *Arabidopsis*, both BPS1 homologues had 349 aa. To understand the relationship between the *Arabidopsis* BPS gene family (*BPS1*, *BPS2,* and *BPS3*) and legume BPS1 homologues, all BPS genes from *Arabidopsis* were used for domain structure analysis. Domain analysis of the BPS genes using *Pfam* revealed the presence of one or two DUF793 domains (PF05633; Figure S2) in all the BPS1 homologues, similar to *Arabidopsis BPS1* genes. However, *Arabidopsis BPS2*, Glyma.10G104200.1 (184 aa), and Glyma.18G129300.1 did not have this domain. BPS3 had a DUF241 domain. The presence of DUF793 domains in *Phaseolus BPS1* homologues confirmed that they were homologues of the *Arabidopsis BPS1* gene. Furthermore, gene structural analysis showed a highly conserved feature of a single intron in the 5′ untranslated region (UTR) in most of the BPS genes (Figure S3).

Conserved motif analysis showed a total of six conserved motifs in BPS1 protein sequences. The motif distribution was similar in all of the BPS1 homologues that contained DUF793 domains. BPS1 homologues of *G.max*, Glyma.10G104200.1, Glyma.18G129300.1, and Lj1g3v2752330.1 did not show all of the motifs that were found in BPS1 homologues containing the DUF793 domain. Further, the motif analysis showed that all the homologues of *Phaseolus*, *Medicago* and *Arabidopsis* had the same motif arrangement in a similar order (Figure S4).

**Figure 1:**
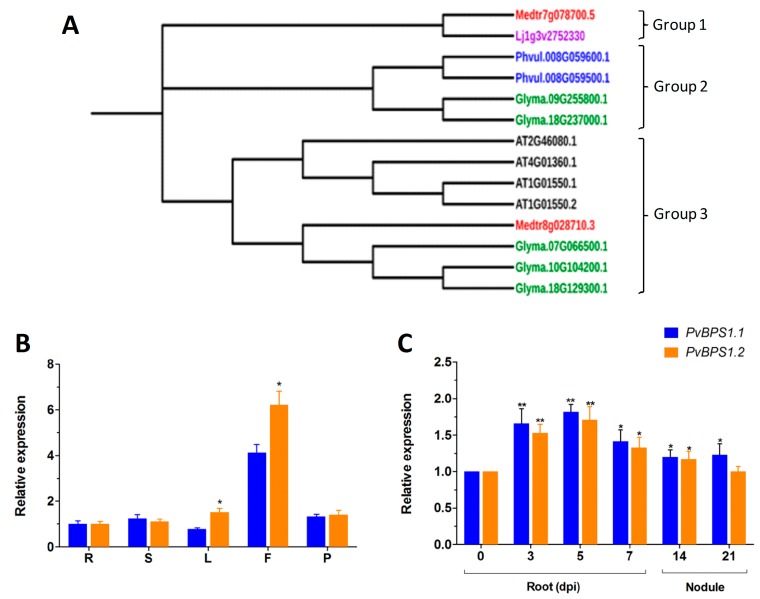
Phylogenetic analysis and expression patterns of *BPS(BYPASS)1*-related genes in wild-type *Phaseolus vulgaris* tissues. (**A**) Protein homologues of BPS1 in *Glycine max*, *P. vulgaris*, *Medicago truncatula*, *Lotus japonicus* and BPS1 (AT1G01550.1 and AT1G01550.2), BPS2 (AT4G01360.1), and BPS3 (AT2G46080.1) of *Arabidopsis thaliana* were constructed using the neighbour-joining (NJ) method with MEGA 7. The phylogenetic tree shows three major groups. Accession numbers are shown in the phylogenetic tree. Bootstrap values are indicated against each branch with 1000 replications. Quantitative real-time PCR (RT-qPCR) analysis of *PvBPS1.1* (Phvul.008G059500) and *PvBPS1.2* (Phvul.008G059600) expression in different vegetative and reproductive tissues (**B**), uninoculated 0, 3, 5, 7, dpi roots, 14- and 21-day-old nodules (**C**). The data are presented as the averages of three biological replicates (*n* > 9), and error bars indicate the means ± standard error of the mean (SEM). The statistical significance of differences between *PvBPS1.1* and *PvBPS1.2* was determined using an unpaired two-tailed Student’s *t*-test (* *p* < 0.05; ** *p* < 0.01). dpi, days post inoculation with *Rhizobium tropici*; R, root; S, stem; L, leaf; F, flower; P, young pod.

Next, phylogenetic analysis of the BPS1 sequences was carried out to understand the phylogenetic relationship among the selected legumes. The results that were obtained using the Neighbour-Joining method in MEGA 7 showed three main groups. Lj BPS1 and Medtr7g078700.5 were in the first group; *Phaseolus* and *G. max* (Glyma.09G255800.1 & Glyma.18G237000.1) formed the second group. The third group had one branch with *Arabidopsis* BPS family genes alone (Figure 1A).

### 3.2. Phaseolus vulgaris BPS1 Express during Root Nodule Symbiosis

The genome of *P. vulgaris* has two copies of the *PvBPS1* gene, *PvBPS1.1* and *PvBPS1.2*. In pairwise alignment, the *PvBPS1.1* amino acid sequence shared 83.5% of its identity with *PvBPS1.2* (Table S2). To understand the expression profiles of *PvBPS1.1* and *PvBPS1.2*, we evaluated the transcript accumulation of vegetative and reproductive organs of wild-type bean plants by quantitative RT-PCR. Transcripts of both *PvBPS1* genes were detected in all of the tested tissues (viz., root, shoot, leaf, flower, and young pod); however, high expression was seen in flower tissues. Among the *PvBPS1* genes, the transcript accumulation of *PvBPS1.2* was found to be significantly higher in leaf and flower tissues when compared to *PvBPS1.1* (Figure 1B). Next, we measured the expression of *PvBPS1* under *Rhizobium tropici* symbiotic conditions. In *Rhizobium*-infected root tissues, the accumulation of both copies of *PvBPS1* transcripts significantly increased in the early stages, i.e., during infection thread formation and cortical cell divisions (3 and 5 dpi), during nodule primordia formation (7 dpi) and detached nodules 14 dpi, when compared to the uninoculated (0 dpi) root tissues (Figure 1C). However, in 21-day-old detached nodules, the promoter expression was found to be induced only in *PvBPS1.1*. Among the *PvBPS1* genes, a slight but not significant difference was observed in the transcript levels between *PvBPS1.1* and *PvBPS1.2* under *Rhizobium* symbiotic condition. Together, these results suggest that *PvBPS1.1* and *PvBPS1.2* are expressed in various organs of the common bean plant and that their expression increases under *P. vulgaris*-*R. tropici* symbiotic conditions.

**Figure 2:**
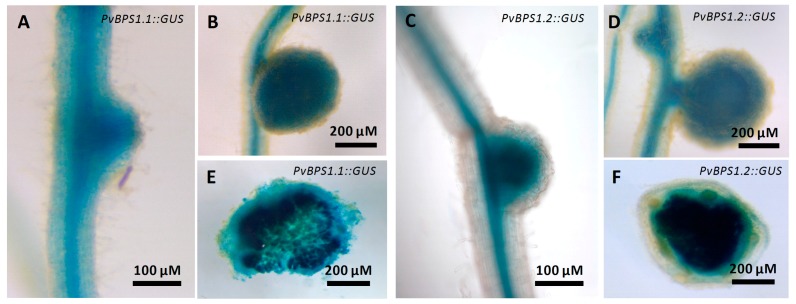
Expression studies of *PvBPS1* promoters with β-glucuronidase reporter in transgenic *P. vulgaris* nodules. Spatial-temporal pattern of *PvBPS1* expression revealed by a promoter: β-glucuronidase (GUS) construct in nodulated transgenic hairy roots after GUS assay. *PvBPS1.1* (Phvul.008G059500) expression in (**A**) nodule primordium at seven days post inoculation (dpi) and (**B**) mature nodules at 18 dpi. *PvBPS1.2* (Phvul.008G059600) expression in (**C**) nodule primordium at 7 dpi and (**D**) mature nodules at 18 dpi. Representative images of nodules (18 days old), (**E**) free hand sections of *PvBPS1.1* and (**F**) *PvBPS1.2*.

We next examined the spatiotemporal activity of *PvBPS1.1* or *PvBPS1.2* promoter-driven GUS expression during nodulation. *P. vulgaris* hairy roots expressing p*PvBPS1.1*::GUS or p*PvBPS1.2*::GUS were inoculated with wild-type *R. tropici*, and the nodulated roots were assayed at different time points to detect GUS activity. *PvBPS1.1* promoter was active in roots and in nodule primordium at 7 dpi (Figure 2A); similarly, the *PvBPS1.2* promoter was found to be active in nodule primordium and in root vasculature (Figure 2C). However, in mature nodules, both *PvBPS1* promoters were expressed at similar levels in nodules and in root vascular tissues (Figure 2B,D). The promoter activity was also detected in central tissues of nodules expressing *PvBPS1.1* or *PvBPS1.2* promoter-*GUS*A fusions (Figure 2E,F). These results show that both *PvBPS1.1* and *PvBPS1.2* promoters are expressed in nodule primordia and in mature nodules of *P. vulgaris*.

### 3.3. Silencing of PvBPS1 Transcripts Affects Shoot and Root Growth

**Figure 3:**
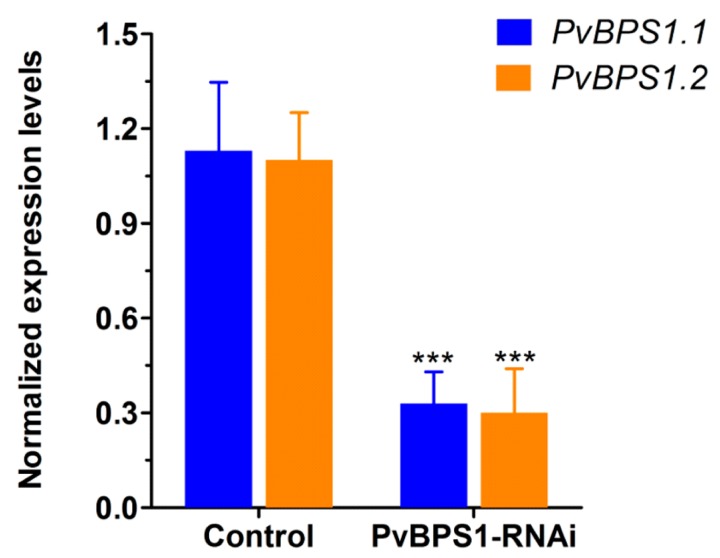
Knockdown of *PvBPS1* genes in *P. vulgaris* transgenic hairy roots. Bean transgenic hairy roots expressing the *PvBPS1-RNAi* construct were analysed by quantitative reverse transcription PCR (RT-qPCR) at 10 days post emergence (dpe) to measure the transcript abundance of the two *PvBPS1* genes viz., *PvBPS1.1* and *PvBPS1.2*. Transcript accumulation was normalized to the expression of the *Ef1α* and *IDE* reference genes. RT-qPCR data are the averages of three biological replicates (*n* > 9). The statistical significance of differences between vector control and interference RNA (RNAi) root samples was determined using an unpaired two-tailed Student’s *t*-test (*** *p* < 0.001). Error bars represent the means ± standard error of the mean (SEM).

There are two *BPS1* genes in the genome of *Phaseolus*, and they share 86% 3′ UTR sequence identity. To silence these *BPS1* genes, a *PvBPS1-RNAi* construct was designed to target a 3′ UTR region comprising 391 bp and cloned downstream of the constitutive 35S promoter into the pTdT-DC-RNAi vector, which co-expresses the tandem dimer tomato (TdT) fluorescent marker that is harboured in the pTdT-DC-RNAi vector. The empty pTdT-DC-RNAi vector was used as the control vector. Next, the *PvBPS1-RNAi* construct was expressed in *Phaseolus* by *A. rhizogenes*-induced hairy roots. Quantitative RT-PCR analysis of the transgenic *PvBPS1-RNAi* roots showed that the transcript levels of both *PvBPS1.1* and *PvBPS1.2* were significantly reduced by ~71% (each) with respect to the transgenic roots of the vector control (Figure 3). These results show that the *PvBPS1-RNAi* machinery was co-silenced by targeting both the transcripts of *PvBPS1.1* and *PvBPS1.2*. Therefore, the nomenclature *PvBPS1*, which refers to both *PvBPS1.1* and *PvBPS1.2*, is used henceforth in the manuscript.

**Figure 4:**
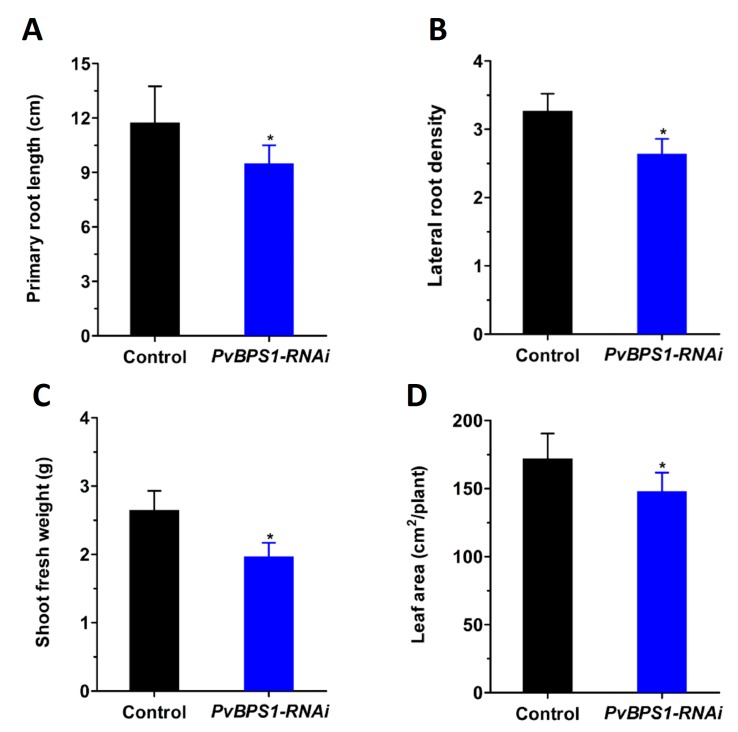
Growth parameters of *PvBPS1-RNAi* composite plants. The *P. vulgaris* composite plants containing transgenic hairy roots were analysed after 10 days post transplantation. (**A**) Primary root length; (**B**) lateral root density in control and *PvBPS1-RNAi* roots; (**C**) Shoot fresh weight and (**D**) total leaf area of composite plants. The data are the averages of three biological replicates (*n* > 18). The statistical significance of differences between control and RNAi root samples was determined using an unpaired two-tailed Student’s *t*-test (* *p* < 0.05). Error bars represent the means ± SEM.

As seen in previous studies in *Arabidopsis*, single (*BPS1*), double (*BPS1*, *BPS2*), and triple (*BPS1*, *BPS2*, *BPS3*) mutants were defective in both root and shoot growth at different intensities [23,24]. Here, to investigate whether the *BPS1* signal affects root and shoot development in *Phaseolus*, we first generated composite plants with hairy roots expressing the *PvBPS1-RNAi* vector. Transgenic roots were carefully selected based on the expression of red fluorescent protein TdT encoded by the vector (Figure S5A–D). Non-fluorescent roots were cut and removed. Control and *PvBPS1-RNAi* plants with equal sizes and numbers of roots and trifoliates were chosen for study to rule out ambiguity. All of the growth parameters were observed at 10 days post transplantation. *PvBPS1-RNAi* lines show a significant decrease in primary root length and lateral root density in comparison with control roots (Figure 4A,B). Furthermore, the composite plants of *PvBPS1-RNAi* produced shoots with less biomass and reduced total leaf area when compared to their control counterparts (Figure 4C,D). This suggests that knockdown of *PvBPS1* significantly reduces both root and shoot growth in *Phaseolus*.

### 3.4. Symbiotic Phenotype in PvBPS1-RNAi Plants

**Figure 5:**
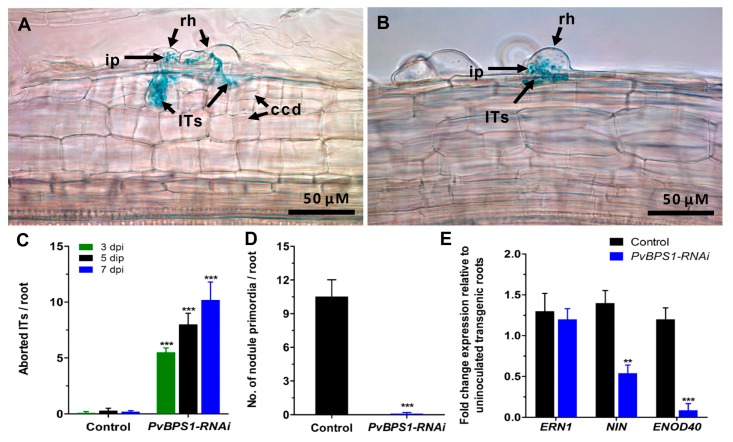
Analysis of phenotype, infection events, and expression profile of early nodulin genes. *Phaseolus* transgenic hairy roots were inoculated with *R. tropici* expressing a β-glucuronidase (GUS) reporter and were stained for GUS at different time points and observed using a light microscope. Representative images showing Its in transgenic roots of (**A**) control and (**B**) *PvBPS1-RNAi* at 3 dpi. Quantitative data showing the average number of (**C**) aborted ITs and (**D**) nodule primordia per transgenic root. (**E**) Quantitative RT-PCR analysis showing the transcript levels of nodulin genes *viz.*, *ERN1*, *NIN* and *ENOD40* in *R. tropici*-inoculated *PvBPS1-RNAi* roots at 3 dpi. Transcript accumulation was normalized to the expression of the *Ef1α* and *IDE* reference genes. RT-qPCR data are the averages of three biological replicates (*n* > 9). The statistical significance of differences between control and RNAi root samples was determined using an unpaired two-tailed Student’s *t*-test (** *p* < 0.01; *** *p* < 0.001). Error bars represent the means ± SEM. rh, root hair; ip, infection pocket; ITs, infection threads; ccd, cortical cell divisions.

Prior to analysing the symbiotic phenotype, the *PvBPS1-RNAi* transgenic hairy roots were examined for any morphological alterations in root hairs. The *PvBPS1-RNAi* roots show no differences in number, length, or position of the root hair cells when compared to control plants that were transformed with pTdT-RNAi empty vector (Figure S6A,B). Next, the *PvBPS1-RNAi* and control lines were inoculated with *R. tropici* expressing GUS reporter to visualize bacterial growth [43]. At 48 h post inoculation, the root hair cells of *PvBPS1* silenced roots show rhizobia-induced root hair deformations that were similar to those seen in the root hair cells of controls (Figure S6C,D). Interestingly, at 3 dpi, the *PvBPS1-RNAi* root hair cells show typical root hair curling; an infection pocket filled with rhizobia bacteria and ITs was observed within the root hair curl. However, these ITs were not associated with cortical cell divisions and were aborted at the base of the root hair cell (Figure 5B), whereas the control hairy roots displayed normal kinetics, including several cell layers of dividing cortical cells that harboured branched ITs (Figure 5A). The *PvBPS1-RNAi* transgenic roots were observed periodically at 3, 5, and 7 dpi, and the aborted IT phenotype persisted at all of the observed time points (Figure 5C). Moreover, these roots were devoid of nodule primordia when compared to the control lines (Figure 5D). The absence of nodule formation was repeatedly observed in *PvBPS1-RNAi* hairy roots of composite plants that were obtained from independent transformation experiments. The transcripts of early nodulin genes, such as *ERN1*, *NIN,* and *ENOD40* showed curious patterns. While *ERN1* expression was not affected in *PvBPS1* silenced roots at 3 dpi, *NIN* and *ENOD40* expression levels were significantly reduced when compared to control roots (Figure 5E). Taken together, the initial signals between the host and *Rhizobium* were not affected by the silencing of *PvBPS1* genes. However, the *PvBPS1* genes are required for the *Rhizobium* infection-associated cortical cell divisions and nodule primordia formation.

### 3.5. Rescue of the Symbiosis Phenotype with Fluridone

**Figure 6:**
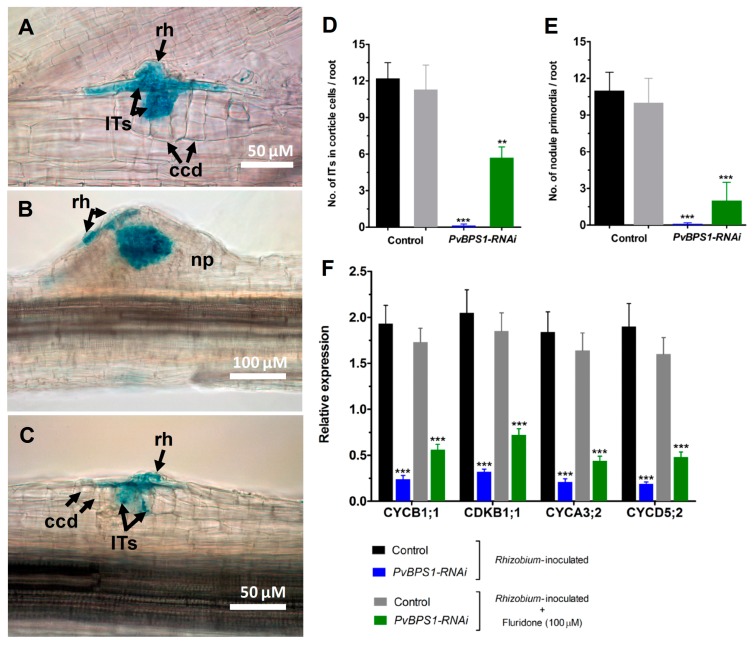
Nodule phenotype rescue by fluridone in *PvBPS1*-silenced roots. Fluridone (100 uM)-treated *Phaseolus* transgenic hairy roots were inoculated with *R. tropici* expressing a GUS reporter and were stained for GUS at different time points and observed using a light microscope. Representative images of transgenic roots showing ITs and ccds in control (**A**), nodule primordia formation in (**B**) control and (**C**) *PvBPS1-RNAi* at 7 dpi. Quantitative data showing the average number of (**D**) ITs found in dividing cortical cells and (**E**) nodule primordia per transgenic root. (**F**) Quantitative RT-PCR analysis of cell cyclins and cyclin-dependent kinase genes in *PvBPS1*-silenced roots under *R. tropici*-inoculated or *R. tropici* plus fluridone-treated conditions. Transcript accumulation was normalized to the expression of the *Ef1α* and *IDE* reference genes. RT-qPCR data are the averages of three biological replicates (*n* > 9). The statistical significance of differences between control and RNAi root samples was determined using an unpaired two-tailed Student’s *t*-test (** *p* < 0.01; *** *p* < 0.001). Error bars represent the means ± SEM. Bar colour description: black—*Rhizobium* inoculated control, grey—*Rhizobium* inoculated + fluridone treated control, blue—*Rhizobium* inoculated *PvBPS1*-RNAi, green—*Rhizobium* inoculated + fluridone treated *PvBPS1*-RNAi. rh, root hair; np, nodule primordia; ITs, infection threads; ccd, cortical cell divisions.

Previous studies in *Arabidopsis* have shown that the synthesis of BYPASS1, a plant-specific protein that negatively regulates the production of a root-derived mobile BPS1, is known to require an intact β-carotenoid biosynthesis pathway [23]. The seedlings of *BPS1* mutants constitutively produced a signal that resulted in a failure to properly establish pro-vascular tissue, the shoot, and the root meristems [22]. The shoot and root phenotype was recovered in *BPS1* mutants when the seedlings were treated with inhibitors of carotenoid biosynthesis pathway [25]. Here, the carotenoid biosynthesis pathway in *PvBPS1*-silenced *Phaseolus* plants were inhibited using 1-methyl-2-phenyl-5-[3-(trifluoromethyl)phenyl]-4[1*H*]-pyridinone (fluridone). First, the roots of wild-type *Phaseolus* plants were irrigated with different concentrations of fluridone (0, 5, 10, 50, 100, and 200 µM) to inhibit carotenoid biosynthesis, which resulted in photobleaching and the eventual death of the plants. As depicted in Figure S7A,B, the 100 µM concentration of fluridone led to 0% plant survival (100% plant mortality) on the 10th day. Next, the same concentration of fluridone was used to treat the *PvBPS1-RNAi* hairy roots that were inoculated with *R. tropici* in order to examine the recovery of the symbiosis phenotype. Typical nodule primordia were formed in the control roots inoculated with either *Rhizobium* (absolute control; Figure S8) alone or *Rhizobium* plus fluridone treatment (Figure 6B). Interestingly, the fluridone-treated *PvBPS1-RNAi* roots show IT progression beyond the root hair cells into the dividing cortical cells (Figure 6C). These rescued ITs and nodule primordial structures were similar to those that were found in controls (Figure 6A,B). Furthermore, the quantitative analysis shows that the average number of ITs that are associated with cortical cells was 5.7 per root in *PvBPS1* silenced lines when compared to 11.3 in controls (Figure 6D). Similarly, at least two nodule primordia per root were found in *PvBPS1* silenced lines, as compared to 10 in controls (Figure 6E). However, as expected, neither infection events were associated with cortical cell divisions, nor nodule primordia were found in *pvBPS1-RNAi* roots inoculated with rhizobia alone (Figure 6D,E).

Since fluridone partially rescued rhizobia-induced cortical cell divisions and nodule primordia formation in *PvBPS1* silenced roots, we next measured the transcript accumulation of cell cyclins and cyclin-dependent kinases using RT-qPCR. Under rhizobia inoculated conditions, the transcripts of CYCB1;1, CYCA3;2, CYCD5;2, and CDKB1;1 were significantly reduced in *PvBPS1*-silenced roots with respect to their controls (Figure 6E). Interestingly, when rhizobia-inoculated *PvBPS1* silenced roots were treated with fluridone, the transcript levels of CYCB1;1, CYCA3;2, CYCD5;2, and CDKB1;1 increased approximately one-fold when compared to the *PvBPS1* silenced roots inoculated with rhizobia alone (Figure 6E). Taken together, our results show that the inhibition of carotenoid biosynthesis in the *PvBPS1*-silenced roots rescues the nodule phenotype, partially in *Phaseolus*.

## 4. Discussion

*Arabidopsis BPS1* mutations led to the accumulation of a mobile signal molecule, *BPS1*, which resulted in severe shoot and root growth defects [22,23,24,25]. The carotenoid biosynthesis pathway is known to be the source of a *BPS* signal that regulates shoot and root meristems through cell cycle arrest at the G_1_ phase [26]. Apart from shoot and root meristem, there is a short-lived meristem activity during the nodule organogenesis of legumes that produce determinate type nodules.

There are several hypotheses proposing that the nodule developmental program is derived from the lateral root development program [44,45,46,47,48,49,50]. Therefore, similar factors could regulate both root and nodule meristems. Among various factors that regulate plant meristems, long-distance signalling molecules, such as plant hormones, are crucial. During plant organogenesis, the balance of long-distance signalling molecules, auxin and cytokinin signalling is critical for generating secondary organs and maintaining the meristematic activity of plant meristems [51].

The recently identified root-derived long-distance signalling molecule SLs is also found to regulate both shoot growth and root nodule development. In the same context, our findings suggest that the BPS signal is a new candidate that plays a critical role in root nodule symbiosis. 

### 4.1. BPS1 is Conserved in Legumes

While identifying *BPS1* homologues, a varying number of *BPS1* genes were encountered in the selected legume species. Phylogenetic analysis showed that the *Phaseolus* and *Glycine* were under one group, irrespective of the conserved motifs in each of these homologues and that the *Arabidopsis* BPS gene family was in the third group with one *Medicago* and two *G. max* homologues. High bootstrap values between *Medicago* (Medtr8g028710.3) and three homologues of *G. max* (Glyma.07G066500.1, Glyma.18G129300.1, and Glyma.10G104200.1) show that they could be paralogues. Peptide motifs play important roles in protein networks. In proteins, domain rearrangements and sequence differentiation are essential for new protein functions [52]. The motif analysis of BPS1 proteins showed that motif distributions were not stable in observed legume species. These differences in motif structures prove that gene rearrangements play a critical role in the BPS1 domain organization of BPS1 proteins, the implications of which need to be investigated.

### 4.2. Ubiquitous Effect of PvBPS1 Silencing in Phaseolus

*PvBPS1* transcripts were detected in all the tested vegetative and reproductive tissues of *Phaseolus*. In *Arabidopsis*, the lack of *BPS1* expression leads to the constitutive accumulation of a graft-transmissible signal, which results in developmental defects in shoots and roots [23]. Here, in composite plants of *Phaseolus*, the hairy roots expressing the *PvBPS1-RNAi* vector showed reductions in primary root length and LR density. At the same time, the untransformed shoot apices also showed developmental defects, such as growing smaller leaves and a reduction in shoot biomass. The shoot phenotype in the present experiment demonstrates once again that the *BPS1* signal is root-derived.

Curiously, *PvBPS1* gene transcripts (*PvBPS1.1* and *PvBPS1.2*) were detected at all of the developmental stages of root nodule development. Specifically, high expression was documented three and five days post inoculation with *Rhizobium*. Furthermore, the promoter expression pattern of the *PvBPS1* genes was found within the central tissues of nodules, which coincides with high transcript accumulation. These data indicate a possible functional role of *PvBPS1* during rhizobial symbiosis in *Phaseolus*.

### 4.3. PvBPS1 Signal Suppresses Cortical Cell Divisions during Root Nodule Symbiosis

Previous reports revealed that a BPS1 signal was a novel developmental signal that functions during embryogenesis and vegetative growth [27]. In the current study, *PvBPS1* silencing in Phaseolus resulted in developmental defects in root and shoot tissues. Unlike *Arabidopsis*, the root hair morphology did not change in Phaseolus, and when these roots were inoculated with *Rhizobium*, normal infection threads grew, which were aborted at the base of root hair cell. However, cortical cell divisions were absent in *PvBPS1*-RNAi plants, indicating that in the absence of the *PvBPS1* transcript, the BPS1 signal accumulates and affects the meristematic activity in the root cortex. The transcripts of early nodulin genes support the observation that early host-*Rhizobium* signalling is not affected. Furthermore, the reduction in transcript accumulation of G_2_/M phase cyclins and CDKs implies cell cycle arrest resulting in the absence of cortical cell divisions.

Furthermore, the partial recovery of the symbiotic phenotype upon treatment with carotenoid biosynthesis pathway inhibitors proves that nodule meristem development is affected by the *BPS1* signal, similar to root and shoot meristems. Nodules are often thought to share some developmental programs with lateral roots. Recent studies confirmed the expression of orthologues of a number of known *Arabidopsis* root meristem regulators in the nodule, among them *MtWOX5*, *MtPLT2,* and *MtBBM*/*PLT4* [53,54,55]. Taken together, these data suggest that the *BPS1* signal could affect all meristem types in plants, including rhizobia-induced cortical cell divisions.

## Supplementary Materials

The following are available online at www.mdpi.com/2073-4425/9/1/11/s1. Figure S1: Multiple sequence alignment of BPS1 proteins; Figure S2: Domain structure of BPS1 homologues; Figure S3: Gene structures of *BPS* homologues; Figure S4: Motif patterns and WebLogo plots of BPS homologues; Figure S5: Transgenic hairy roots expressing red fluorescence in *Phaseolus*; Figure S6: *Phaseolus* transgenic hairy roots showing root hair morphology; Figure S7: Fluridone dose-response curve (survival) in wild-type *P. vulgaris* plants; Figure S8: Young nodule on transgenic control root. Table S1: Primer sequences of *Phaseolus vulgaris* genes used for cloning and RT-qPCR; Table S2: Percent amino acid sequence identity of BPS1 genes.
