# Supplementary material for: In BPS1 Downregulated Roots, the BYPASS1 Signal Disrupts the Induction of Cortical Cell Divisions in Bean-Rhizobium Symbiosis
Source: Genes (Basel). 2018 Jan 3;9(1):11. doi: 10.3390/genes9010011 (PMC5793164; doi:10.3390/genes9010011)
Supplement: Supplementary file 1 [file genes-09-00011-s001.zip › Table S1.docx]

**Table S1:** Primer sequences of *Phaseolus vulgaris* genes used for cloning and RT-qPCR.

| Gene (IDs) | | Oligonucleotide sequence |
| --- | --- | --- |
| *BPSi-RNAi* | F | 5′- AG CTT TCA CAG GGA CTA GAT C-3′ |
|  | R | 5′- CAA ACT TCA ATG AGC ACA GGT AAC-3′ |
| *pPvBPS1.1*  (Phvul.008G059500) | F | 5′-CAC GTG GGA AAA GCA CCA ACG-3′ |
|  | R | 5′- GAA AGT CAA CAT CTG TCG TCA C-3′ |
| *pPvBPS1.2*  (Phvul.008G059600) | F | 5′- GGT ACC ACG TGT TCA ATA TTC-3′ |
|  | R | 5′- GGA AGT AGT AAG ATA ACA CAT TGA TG-3′ |
| *PvEf1α*  (Phvul.004G075100) | F | 5′- GGT CAT TGG TCA TGT CGA CTC TGG-3′ |
|  | R | 5′- GCA CCC AGG CAT ACT TGA ATG ACC-3′ |
| *PvIDE*  (Phvul.001G133200) | F | 5′- GCA ACC AAC CTT TCA TCA GC-3′ |
|  | R | 5′-AGA AAT GCC TCA ACC CTT TG-3′ |
| *PvBPS1.1*  (Phvul.008G059500) | F | 5′-CAC GTG GGA AAA GCA CCA ACG A-3′ |
|  | R | 5′-GAT GAA GGG AGG TGA TGA TG-3′ |
| *PvBPS1.2*  (Phvul.008G059600) | F | 5′-AAC TCT GGT ACA TAC CGA TGT A-3′ |
|  | R | 5′-AAC ATC ACA GAT ATA TAT TTC-3′ |
| *PvRIP1*  (Phvul.001G111800) | F | 5′-GTC GAA TCT CGC CTT GT-3′ |
|  | R | 5′-GGC CCT GTT GTA TCT TGT GC-3′ |
| *PvERN1*  (Phvul.001G111800) | F | 5′-GGA GCT GTC TTT GAT CGT TTT CC-3′ |
|  | R | 5′-CAA ATT CAG AAA GCT CCA AGT CAG C-3′ |
| *PvENOD40*  (Phvul.002G064200) | F | 5′-AGT TTT GTT GGC AAG CAT CC-3′ |
|  | R | 5′-TAA GCA CAA GCA AAC TGT TG-3′ |
| *PvNIN*  (Phvul.009G115800) | F | 5′-GGG GAT TCA GAG ATT TGC AG-3′ |
|  | R | 5′-AAC CCA CTC TTG AGC ATC GT-3′ |
| *PvCycA3-2*  (Phvul.009G069000) | F | 5′-GAA GAT TAG TGA TGG AGT G-3′ |
|  | R | 5′- CCC TAT CAA GAT AGG TTA A-3′ |
| *PvCycD5-2*  (Phvul.009G071400) | F | 5′-GGG AAG GGA CAG GGC TAG A-3′ |
|  | R | 5′-GAC ATT CTT GAT TAG TTC TAG C-3′ |
| *PvCycB1*  (Phvul.008G203300) | F | 5′-AGT GTT GTC AAG TGC TTT GCT GGA G-3′ |
|  | R | 5′-GGA TTG CGC CAA AAA CCT AGT-3′ |
| *PvCdkB1-1*  (Phvul.008G253500) | F | 5′-GCC AAG GCA GCA CTT GAA C-3′ |
|  | R | 5′- GGC AAC CAT GAA CTG CC-3′ |
