# Supplementary material for: In BPS1 Downregulated Roots, the BYPASS1 Signal Disrupts the Induction of Cortical Cell Divisions in Bean-Rhizobium Symbiosis
Source: Genes (Basel). 2018 Jan 3;9(1):11. doi: 10.3390/genes9010011 (PMC5793164; doi:10.3390/genes9010011)

**Table S2:** Percent amino acid sequence identity of BPS1 genes. Multiple sequence alignment of BPS1 amino acids of *Arabidopsis* and legumes.


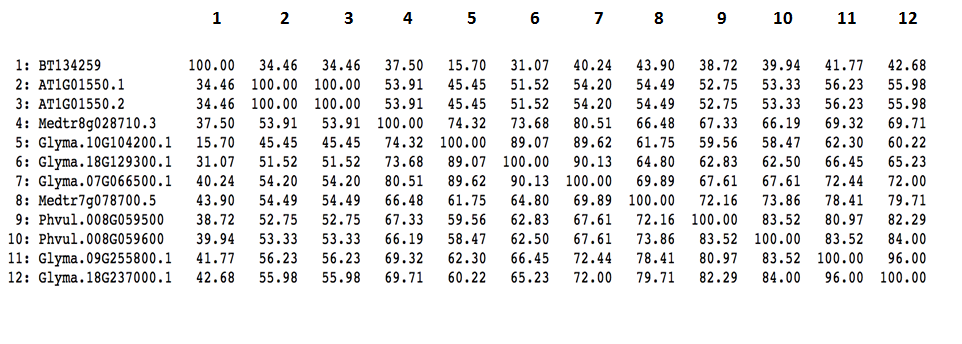

Supplement: Supplementary file 1 [file genes-09-00011-s001.zip › Table S2.docx]
