# Supplementary material for: In BPS1 Downregulated Roots, the BYPASS1 Signal Disrupts the Induction of Cortical Cell Divisions in Bean-Rhizobium Symbiosis
Source: Genes (Basel). 2018 Jan 3;9(1):11. doi: 10.3390/genes9010011 (PMC5793164; doi:10.3390/genes9010011)
Supplement: Supplementary file 1 [file genes-09-00011-s001.zip › Figure S1.doc]

**Figure S1**

```
BT134259           1 MSRPQD--PPRSFFPFGNPFRMI-APK-GTKLSPQLLEVICNFEAKLEERMKKLIPKSKD
AT4G01360.1        1 MSQQRT--RSR------NPFKILFTKKNNARLSPKLLSLLNNFETNLTVSIRELVPKDKN
Medtr8g028710.3    1 MSRSQDPHPNRSFFPFGNPFRTK-SHKGGSHMSPQLLAVLHGFEATLAERLRKLMPKSKD
Glyma.10G104200    1 ------------------------------------------------------------
Glyma.18G129300    1 ------------------------------------LILQTNY----------LVPKSKD
Glyma.07G066500    1 MSRSQDPHPSRSFFPFGNPFRMI-SPK-GSRMSPQLLAVLQAFEATLAERLKNLMPKSKD
Medtr7g078700.5    1 MSRPQD--PPRSFFPFGNPFRMI-APK-GTKLPPQLLAVLSDFEATLEERLKNLIPKSKD
Phvul.008G05950    1 MSRAHD--INRSFFPFGNPFRMK-SPR-GTKITSQLLLVSQAFEASLEERLKKLIPESKD
Phvul.008G05960    1 MSRAQD--GNRSFFPFGNPFRMI-SPK-GPKITSQLLSVLQAFEATLEERLKKLIPESKD
Glyma.09G255800    1 MSRAQD--ANQSFFPFGNPFRMI-SPK-SPKISSQLLLILHAFEATLEERLKKLIPKSKD
Glyma.18G237000    1 MSRAQD--ANRSFFPFGNPFRMI-SPK-GPKISSQLLLILHAFEATLEERLKKLIPKSKD
AT1G01550.1        1 MARPQD--PPRGFFPFGNPFKNL-SSK-NSVLSSKLLPLLNNFETNLASSISKLVPKEKS
AT1G01550.2        1 MARPQD--PPRGFFPFGNPFKNL-SSK-NSVLSSKLLPLLNNFETNLASSISKLVPKEKS
AT2G46080.1        1 MSRPQD--PPRGFFPFGNPFRML-SSK-GSDLSPWLLSLLNGFELRLEERLKKLMPKNKD
consensus          1 msr qd  p rsffpfgnpfrmi spk gtkls qll vlngfeatleerlkklipkskd


BT134259          57 EILCLSWMTSAMQALCESHNDIKTLIDNLELPVHDWDEKWIDVYLDISVKLLDVCIAFSS
AT4G01360.1       53 DIVSVSWMIQAMQSLCETHKSIRTLVKDLEVPVSDLEENFIYIFSDFSLK-RELCNAFTS
Medtr8g028710.3   60 EILSLAWMTLAMKSLCETHNDIRTLITDLELPVSVWDDKWVDVYFDISTKLLDICNIFSS
Glyma.10G104200    1 ------------------------------------------------------------
Glyma.18G129300   15 EILTLSSMTLAMKSLCESHNDIWTLMTALELPVSDWEGKWIDVYFDISSKLLEICNGFSS
Glyma.07G066500   59 EILTLSWMTLAMKSLCESHNDIRTLMTALELPVSDWEDKWIDVYLDISSKLLDICNAFSS
Medtr7g078700.5   57 EVVSLSWMTSAMQALCESHNDIKSLMTNLELPVTDWDEKWIDVYLDISVKLLDICIAFSS
Phvul.008G05950   57 EILSLSWMTFAMRALSESYDDIKTLVTELELPVNDWDEKWIDVYFDISVKLLDICNVFSS
Phvul.008G05960   57 EILSLSWMTLAMQVLCESHNDIKTLITDLEMPVNDWDEKWIDVYFDISMKLLDMCNAFSS
Glyma.09G255800   57 EILSLSWMTLAMQSLCESHNDIKTIITELELPVHDWDEKWIDVYLDISVKLLDICIAFSS
Glyma.18G237000   57 EILSLSWMTLAMQALCESHNDIKTLITELELPVHDWDEKWIDVYLDISVKLLDICIAFSS
AT1G01550.1       57 DILTVSWMKQAMESLCETHNGIKTLITDLELPVSDWEDKWVDVYLDISVKLLDLCNAFSS
AT1G01550.2       57 DILTVSWMKQAMESLCETHNGIKTLITDLELPVSDWEDKWVDVYLDISVKLLDLCNAFSS
AT2G46080.1       57 DILTLSWMKLAMESLCETHKNINTLITDLQLPVSDWEEKWVDVYLNISVRLLDLCNAFSS
consensus         61 eilslswmtlam slceshndiktlitdlelpvsdwdekwidvyldisvklldicnafss


BT134259         117 ELSRLNQGQLLLQCALHHLDESSQSSLFGPVLHLMVGGSILVLKILGLRNV-AIFWRISR
AT4G01360.1      112 EIYHLEHGNLLLKFAFSKLETNSGN-------ILQRWNQHMVSKNPSIENWGAVLSRLVE
Medtr8g028710.3  120 ELSRLNQGNLPLKCALHNLGPASSKSFVRACSLLDDWRRHINAKNPRIEKCSTILDGLVG
Glyma.10G104200    1 -----------------------------------------------------MLDNLVG
Glyma.18G129300   75 ELSRLNQGNLPLKY------SASSKQYLQACSLLDDWRQHVSSRNPRIEKCSSMLDNLVG
Glyma.07G066500  119 ELSRLNQGNLPLKCALHNLDSASSKQYLRACSLLDDWRQHVSSRNPRIEKCSSMLDNLVG
Medtr7g078700.5  117 ELSRLNQGRLLLQCTLHHFGSFSSDQLFQAYSSLDGWRQHIGSKNPRIENCGSVLDNLAG
Phvul.008G05950  117 ELSRMSQGHLLLQCALHNLGSSSSEQYGRACSSLDGWKQHIGSGNPRIEKCRNILDGLLG
Phvul.008G05960  117 EISRMSQGHLLLQCALHNLGSSSSEQYDRACSSLDGWKQHICSENVRIEKCGSILDGLLA
Glyma.09G255800  117 ELSRLNQGHLLLQCALHNLGSSSSEQYVRACSSLDGWKQHIGSGNPRIEKCGSILDDLLR
Glyma.18G237000  117 ELSRLNQGHLLLHCALHNLGSSSSEQYVRACSSLDGWKQHIGSGNPRIEKCGSILDNLLR
AT1G01550.1      117 ELTRLNQGHLLLQFALHNLEANSPQNLSKAQSSLDSWKQHIVSKNPRIENCRAILSSLVQ
AT1G01550.2      117 ELTRLNQGHLLLQFALHNLEANSPQNLSKAQSSLDSWKQHIVSKNPRIENCRAILSSLVQ
AT2G46080.1      117 ELTRLNQGDLFLKCVLHNLQSDSGEKYLQARSSLDSWRQHVNTNNPRIENCRAVLDSLVK
consensus        121 elsrlnqgrlllqcalhnl s ss qy racssld wrqhi sknpriekc tild lvg


BT134259         176 DHLIF-------RLKSQLKGR-FCRLFMERRQYLFAVCLLRFFQVLQRICLTWMWLTCI-
AT4G01360.1      165 SMDHHHHISKKGKKKYSAEGKVLLRVLYGVKVKTLYIFSV-FDASFSGSSKNLFYLTIPK
Medtr8g028710.3  180 SLDLP-------KVKNSAKGKALMQAMYGVKVETAFVCSV-FSAAFSGSSKKLLDLDVP-
Glyma.10G104200    8 SLNLP-------KVKNSTKGKVLMQAMYGVKVETVFICRV-FTAAFSGSSKKLTNLNAV-
Glyma.18G129300  129 SLDLP-------KVKNSTKGKVLMQAMYGVKVKTVFICRV-FTATFSGSSKKLSNLNVA-
Glyma.07G066500  179 SLDLP-------KVKNSAKGKVLMQAMYGVKVETVFICRV-FTAAFSGSSKKLSDLNVA-
Medtr7g078700.5  177 SSDLP-------KVKKSAKGKVLMQAIYGVKALTVFVCGV-FATAFSGSTKNLMDMDVA-
Phvul.008G05950  177 SLDLP-------KVKKSAKGKVLMQAMYGVKVLTVFVCSV-FAAVFSGSTKNMLDLDVA-
Phvul.008G05960  177 SLDLP-------KVKKLAKGKVLVQAMYGVKVLTVFVCSV-FAASFSRSTKNMLDLNVA-
Glyma.09G255800  177 SLDLP-------KVKKSAKGKVLMQAMYGVKVQTVFVCSV-FAAAFSGSTKNMSDLNVA-
Glyma.18G237000  177 SLDLP-------KVKKSAKGKVLMQAMYGVKVQTVFLCSV-FAAAFSGSTKNMSDLDVA-
AT1G01550.1      177 TLNLP-------KVKNSAKGKVLMRALYGVKVKTLYISGV-FAAAFSGSSQNLMYLTVS-
AT1G01550.2      177 TLNLP-------KVKNSAKGKVLMRALYGVKVKTLYISGV-FAAAFSGSSQNLMYLTVS-
AT2G46080.1      177 SLSLP-------KVKNSPKGKVLMRAFYGVKVQTVYICSV-FTAAWSDSTKDLFDLPVS-
consensus        181 sldlp       kvKnsakGkvlmqamygvkv tvficsv Faaafsgssknlmdl va


BT134259         227 ------LGLQLLKVCRMLMRKLELDFQVGDWSASWKRLIPLRNYILLFKVL----M---I
AT4G01360.1      224 EMEELPWVQAFMELQNTINPEIKNTFLSDGFTVIKDLEAVETGVKKLYTAVQEGSV---P
Medtr8g028710.3  231 --DMHSWAPAFISLQNLVNEEIRVRLSGGKFSVLIELEAVDAVVKELYPTIQGG-VNTED
Glyma.10G104200   59 --DIHSWDLDFRRLQNLVNEEIRVRFSGGKFTVLNELEAVDASVKILYPTIQTG-V---D
Glyma.18G129300  180 --DIHSWAPDFRRLQNLVNEESRVRFSGGKFTVLNELEAVDASVKILYPTIQAG-V---D
Glyma.07G066500  230 --DIHSWAPDFRRLQDLVNEEIRVRFSGGKFTVLNELEAVDASVKILYPTIQAG-V---D
Medtr7g078700.5  228 --DVYSWAPTFKGLQNLVNEEIRVRFSSGRFTVLNELEAVDLSVRELYPIIQGV-V---D
Phvul.008G05950  228 --DIYSWASSFKSLQDVVNEEIRGRFSSGRFTVMNELESVDSSVKELYPIIQGV-V---D
Phvul.008G05960  228 --DVYSWPPTFKRLQNLVNEEIRVRFSCGRFAVLNELESVDSSVKELYPIIQGV-V---D
Glyma.09G255800  228 --DVYSWAPTFKSLQDLVNEEIRVRFSSGRFTILNELEVVDSSVKELYPIIQGI-A---D
Glyma.18G237000  228 --DVYSWAPTFKSLQDLVNGEIRVR--SGRFTILNELEVVDSSVKELYPIIQGV-S---D
AT1G01550.1      228 --NELPWAQSFMEVQNTMNAEIKNIFLSDGLTVLKELEAVASGVKKLYPAIQQGSI---D
AT1G01550.2      228 --NELPWAQSFMEVQNTMNAEIKNIFLSDGLTVLKELEAVASGVKKLYPAIQQGSI---D
AT2G46080.1      228 --EKPLWAKVFTDMQSVVNAEIRDMLSSGRTTILKELESVDASVEKLYPMIQDG-V---D
consensus        241   dm swaptfr lqnlvneeirvrfssgkftvlneleavdtsvk Lypviqgg v   d


BT134259         274 PLSQNHLSKLLRSL-AEKQRSSHKDIFLQRKWMAFSKSCLV---VMLCYLIG----QVQP
AT4G01360.1      281 ILLVEPLEKSVIE-------LSERFELVSKETRCLSKMVISARDALLESLWTKCAEELGV
Medtr8g028710.3  288 KVEQESHLKTVEELGVAAEKLSQGMDLLAKGVDGFFQAVLTSRDTLLSSLRF------GK
Glyma.10G104200  113 TIEIEWLLKTVEELRAGAEKLSQGNNLLAKGVDGFFEAVMTSRDTLLSSVRF------DK
Glyma.18G129300  234 TIEIEWLVKTVEELHAGAEKLSQGNDLFAKGVDGFFEAVMTSRDTLLSSMRF------DK
Glyma.07G066500  284 TVETEWLVKTVEELRAGAEKLSQGIDLLAKGVDGFFQAVMTSRDTLLSSVRF------DK
Medtr7g078700.5  282 TIETESLAKTVEKLGRATENFSQGLDLLTKEVDGFFQVVLSGRDALLSNLRS------GA
Phvul.008G05950  282 PVKMESHTKIVEELGKSTEKLSQGLDLLAKEVDSFFQVVLAGRDALLSSLRS------GA
Phvul.008G05960  282 PIKMESLPGIVGELGRSTAKLSQGLDLLTKEVDSFFHVVLTGRDALLSNLRS------GA
Glyma.09G255800  282 TIETESLAKIVGELSRATEKLSQGLDLLTKEVDSFFQVVLTGRDTLLSSLRS------GA
Glyma.18G237000  280 AIETESLAKIVGELGRATEKLSQGLDLLTKEVDSFFQVVLTGRDALLSSLRS------GA
AT1G01550.1      283 PISLQPLKDSVTE-------LSNGIDLVSKEVDCFFKILLSGRDTLLENLRSMGASTLQA
AT1G01550.2      283 PISLQPLKDSVTE-------LSNGIDLVSKEVDCFFKILLSGRDTLLENLRSMGASTLQA
AT2G46080.1      282 PVEVEFFKDNVMELGTQAEKLSQGLDQLLEEVDSFFKLTLRGRDVLLCNLRS------SD
consensus        301  ieve l ksv el  a eklSqgmdllskevd ff mvltgrdtlLsslrs      ga


BT134259         326 SMTAFWGVTEMPKQSIX
AT4G01360.1      334 TLPTMVKSVNIEEKS--
Medtr8g028710.3  342 TVNDRVVGRNLDQQVVY
Glyma.10G104200  167 IVNDRSLGRNRDMQLVH
Glyma.18G129300  288 TVNDHSPGRNRHMQVVH
Glyma.07G066500  338 TVNDRSPGRNRDMQVVH
Medtr7g078700.5  336 TVNDHILRGNKDAQVVN
Phvul.008G05950  336 TVIHSNMGVNRNAQIVS
Phvul.008G05960  336 TVIPSNMGGNRNAQIVN
Glyma.09G255800  336 TFIDSIQAGNRDAQIVN
Glyma.18G237000  334 TFIDSIQAGNRDAQIVN
AT1G01550.1      336 TSPKKAAGKNYRGF---
AT1G01550.2      336 TSPKKAAGKNYRGF---
AT2G46080.1      336 SISGNAVGEDVD-----
consensus        361 tv dr  g nrdaqvv


```

**Figure S1:** Multiple sequence alignment of BPS1 proteins. Amino acid sequence alignment of the BPS1 protein with the deduced amino acid sequences from the legume species *G. max, P. vulgaris, M. truncatula, L. japonicus* and *Arabidopsis*. Multiple sequence alignment was performed using T-Coffee and alignment output was generated using Boxshade version 3.21. Black boxes signify identical residues within a column consensus and gray boxes signify similar residues within a column consensus. In the consensus row, capital letters indicate sequence identity between all the species and lowercase letters indicate sequence identity between *Phaseolus* (Phvul.008G059500) and other species.
